# Excess of health care use in general practice and of comorbid chronic conditions in cancer patients compared to controls

**DOI:** 10.1186/1471-2296-13-60

**Published:** 2012-06-19

**Authors:** Lea Jabaaij, Marjan van den Akker, François G Schellevis

**Affiliations:** 1 NIVEL (Netherlands Institute for Health Services Research), PO Box 1568, Utrecht, 3500 BN, The Netherlands; 2 Caphri: School for Public Health and Primary Care, Department of General Practice, Maastricht University, Maastricht, The Netherlands; 3 Department of General Practice, Katholieke Universiteit Leuven, Leuven, Belgium; 4 Department of General Practice and Elderly Care Medicine/EMGO Institute for Health and Care Research, VU University Medical Centre, Amsterdam, The Netherlands

**Keywords:** Neoplasms, Cancer, Primary health care, General practitioner, Follow-up, Comorbidity

## Abstract

**Background:**

The number of cancer patients and the number of patients surviving initial treatments is expected to rise. Traditionally, follow-up monitoring takes place in secondary care. The contribution of general practice is less visible and not clearly defined.

This study aimed to compare healthcare use in general practice of patients with cancer during the follow-up phase compared with patients without cancer. We also examined the influence of comorbid conditions on healthcare utilisation by these patients in general practice.

**Methods:**

We compared health care use of N=8,703 cancer patients with an age and gender-matched control group of patients without cancer from the same practice. Data originate from the Netherlands Information Network of General Practice (LINH), a representative network consisting of 92 general practices with 350,000 enlisted patients. Health care utilisation was assessed using data on contacts with general practice, prescription and referral rates recorded between 1/1/2001 and 31/12/2007. The existence of additional comorbid chronic conditions (ICPC coded) was taken into account.

**Results:**

Compared to matched controls, cancer patients had more contacts with their GP-practice (19.5 vs. 11.9, p<.01), more consultations with the GP (3.5 vs. 2.7, p<.01), more home visits (1.6 vs. 0.4, p<.01) and they got more medicines prescribed (18.7 vs. 11.6, p<.01) during the follow-up phase. Cancer patients more often had a chronic condition than their matched controls (52% vs. 44%, p<.01). Having a chronic condition increased health care use for both patients with and without cancer. Cancer patients with a comorbid condition had the highest health care use.

**Conclusion:**

We found that cancer patients in the follow-up phase consulted general practice more often and suffered more often from comorbid chronic conditions, compared to patients without cancer. It is expected that the number of cancer patients will rise in the years to come and that primary health care professionals will be more involved in follow-up care. Care for comorbid chronic conditions, communication between specialists and GPs, and coordination of tasks then need special attention.

## Background

In general, cancer is a disease of the old age. Highest prevalence rates of almost all cancers are found among persons older than 55 years of age. For example, while this age group comprises only 20% of the population in the United States, over 80% of the invasive cancers are found in this group [1]. Therefore, with an ageing population (for figures of the Netherlands see [2]), it is expected that the number of cancer patients will increase substantially. Diagnosis, initial treatment of cancer and follow-up care is for the larger part provided by specialists working in secondary care [3,4]. With an increasing number of patients with cancer and an increasing number of cancer patients surviving for a longer period of time a reorganization of health care will be necessary. New roles for general practitioners (GPs) and other primary care professionals in cancer care might emerge, for example in follow-up care.

Anticipating these future developments, it is interesting to know what care is currently delivered to cancer patients by GPs. In literature, few studies are found which focus on health care use of cancer survivors in primary care. Roorda et al. [5] reported higher health care utilisation rates in general practice for breast cancer patients during the year after diagnosis compared to the control group. This is in line with the results of a British study [6]: the year after their diagnosis of breast cancer the contact rate of patients with their GP more than doubled compared to the year preceding their diagnosis. Two years after their diagnosis, the contact rate had not yet returned to the baseline level. In a group of long-term survivors of Hodgkin's disease, French researchers found a slight increase in the number of visits to a general practitioner compared to matched control patients [7]. A study that did not match these results concluded that the consultation rates of cancer patients in general practice did not increase when GPs took care for discharged long-term cancer survivors [8]. However, these patients were voluntarily discharged from secondary care and might not be a representative sample of long-term cancer survivors.

We studied health care use in general practice of patients with cancer during the follow-up phase. Did they use more health care in general practice, compared to patients without cancer? Because many cancer patients are older than 65 years, we also focussed on the presence of comorbid chronic conditions and whether these conditions influenced health care use in general practice.

## Methods

### Design

To analyse health care use of cancer patients in general practice we used routinely collected data from the Netherlands Information Network of General Practice (LINH). We compared the health care use of cancer patients during the follow-up phase with age and gender matched control patients from the same practice in a retrospective cohort study.

### General practices

The Netherlands Information Network of General Practice (LINH) is a representative network consisting of 92 general practices from all over the Netherlands with about 350,000 enlisted patients. In the Netherlands, every inhabitant is listed with a general practitioner. The LINH database holds longitudinal data on GP care, including patient contacts, referrals, prescription medicines, and relevant health problems. Health problems are coded by the GP, using the International Classification of Primary Care (ICPC) [9]. The database is used for health services research and quality of care research. For more information on the network see http://www.linh.nl.

### Patients and controls

**Figure 1:**
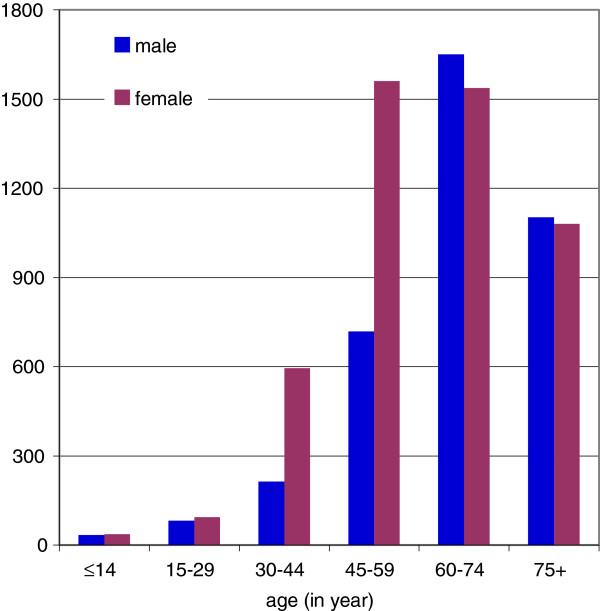
Number of patients with cancer by gender and age group (absolute numbers; males n=3800, females n=4903).

Based on ICPC-codes [see paragraph below] we selected all patients for whom a diagnosis of cancer was recorded between January 1, 2001 and December 31, 2007. This resulted in 8,703 cancer patients, 44% males and 56% females for whom health care data were available on a minimum of 30 days during the follow-up phase (see further). The age distribution of the cancer patients is shown in Figure 1.

Cancer patients were matched for age (in five year intervals) and gender with patients without a diagnosis of cancer, enlisted in the same general practice. We could include 8,672 control patients (data not shown).

### Type of cancer

For 69.2% of all selected patients with cancer (N=8,703) one of the following cancer types was recorded: breast cancer (ICPC code X76, 27.7% of all patients), colon/rectal cancer (D75, 13.6% of all patients), prostate cancer (Y77, 13.5% of all patients), lung/bronchus cancer (R84, 9.3% of all patients) and cancer of the bladder (U76, 5.1% of all patients).

For 70.0% of the female patients with cancer (N=4,903) one of the following cancer types was recorded: breast cancer (X76, 49.3% of the women), colon/rectal cancer (D75, 11.8% of the women), and malignancies of female reproductive organs (X77, 8.9% of the women).

For 61.2% of the male patients with cancer (N=3,800) one of the following cancer types was recorded: prostate cancer (Y77, 30.9% of the men), colon/rectum cancer (D75, 15.8% of the men) and lung/bronchus cancer (R84, 14.4% of the men).

Other cancer types with low numbers of patients included the following ICPC coded cancer types: A79 (malignancy not otherwise specified (NOS)), B72 (Hodgkin's disease/lymphoma), B73 (leukaemia), B74 (malignant neoplasm blood other), D74 (malignant neoplasm stomach), D76 (malignant neoplasm pancreas), D77 (malignant neoplasm digestive system other/NOS), N74 (malignant neoplasm nervous system), R85 (other malignant neoplasm respiratory), S77 (malignant neoplasm of the skin, excluding squamous and basal cell carcinoma), T71 (malignant neoplasm thyroid), U75 (malignant neoplasm of kidney), U77 (malignant neoplasm urinary other), W72 (malignant neoplasm related to pregnancy), X75 (malignant neoplasm cervix uteri), Y78 (malignant neoplasm male genital other).

### Follow-up phase and observation period

We studied use of health care in general practice of cancer patients during the follow-up phase. We defined this phase as starting after the end of the initial treatment and ending before the terminal phase started. Both the initial treatment phase and the terminal phase were excluded, because during these periods patients might have special health care needs that are not representative for the remainder of this period. We set the end of initial treatment to be six months after the first recording of the diagnosis in the electronic medical record (EMR) in the practice and the start of the terminal phase on three months before death or the last registration in the EMR. For reasons of reliability we included only those patients of whom an observation period of at least 30 days was available.

All health care data recorded in the EMR between January 1, 2001 and December 31, 2007 were included in the analyses. Of all patients 78% was still alive at the end of the observation period, 17% had died, and 5% of the patients left the practice because they moved. The length of the observation period differs for every patient, depending on the date of diagnosis. For the most frequently represented types of cancer we found the following median lengths of the observation period: breast cancer (X76) 644 days, colon/rectal cancer (D75) 556 days, prostate cancer (Y77) 598 days, lung/bronchial cancer (R84) 432 days, and cancer of the bladder (U76) 612 days.

### Comorbid chronic conditions

For every patient the presence of comorbid chronic conditions was determined using ICPC coded diagnoses recorded during contacts, prescriptions or referrals. We used the classification for comorbid chronic conditions as described by Knottnerus et al. [10]: sensory conditions (including ear and eye conditions), heart disease, musculoskeletal system, neurological-movement conditions, neurological-pain conditions, psychiatric conditions, respiratory conditions, skin conditions, endocrinological conditions, and urogenital conditions.

### Health care use

The EMR encompasses routinely recorded data for every patient enlisted in the practice, including contacts with the practice, morbidity, referrals to other health care providers and drugs prescribed. The total number of contacts with general practice includes face to face consultations with the GP, home visits, telephone contacts for prescriptions, vaccinations, email contacts, and administrative activities. For all data recorded during the observation period numbers were recalculated to a one year’s period.

In the Netherlands the GP functions as the ‘gatekeeper’ of care, meaning that patients need a referral for specialist health care or for other primary health care workers [11]. The number of referrals can be seen as an indication of the ability of GPs to deal with requests for treatment themselves. We calculated the mean number of new referrals per patient per year.

### Analyses

Cancer patients were compared with age- and gender-matched control patients with regard to the number of contacts with general practice, diagnoses of comorbid conditions, rate of prescribing, and the referral rate in a year.

Furthermore, we studied the influence of having a comorbid chronic condition on health care use. The group of cancer patients without comorbid chronic conditions was compared with the matched control group without comorbid chronic conditions while the group of cancer patients with comorbid chronic conditions was compared with the matched control group with comorbid chronic conditions.

We calculated mean figures for the number of contacts with the practice, prescriptions and referrals, and tested for statistical significance of the differences between the two groups using univariate variance analyses (Student’s *t*-test). Because of our large sample size, p=0. 01 was set as cut-off value for statistical significance.

## Results

### Health care use in general practice

During the period observed, cancer patients used more care in general practice as compared to matched controls without cancer (Table 1). They had more contacts in the general practice in a year, a higher number of face to face consultations with the GP, more home visits, more prescriptions, and more referrals to secondary specialist care.

**Table 1:** Health care use of cancer patients (n=8,703) compared to matched controls (n=8,672) (mean number and standard error of the mean (SEm) of contacts, prescriptions and referrals per patient in a year)

	**cancer patients (mean and (SEm))**	**patients without cancer (mean and (SEm))**
contacts with general practice	19.5 (.2)*	11.9 (.1)
- face to face consultations	3.5 (.05)*	2.7 (.04)
- home visits	1.6 (.06)*	.40 (.02)
prescriptions	18.7 (.2)*	11.6 (.2)
referrals	0.56 (.1)*	0.42 (.1)

* difference between cancer patients and patients without cancer: p<0.01.

The higher health care use of cancer patients was found both in males and in females (Table 2). Health care use of cancer patients older than 30 years of age was higher compared to the matched control group. However, cancer patients younger than 30 years of age did not differ from age-matched controls in the number of face to face consultations with the GP, the number of home visits and referrals. But compared to the control group they did have a higher total number of contacts with the general practice, caused by more telephone contacts and/or, email contacts and/or administrative activities in the electronic medical record.

**Table 2:** Health care use of cancer patients by gender and age group (n=8,703) (mean number of contacts, prescriptions and referrals per patient in a year)

	**gender**		**age group (year)**					
	**male**	**female**	**≤ 14**	**15-29**	**30-44**	**45-59**	**60-74**	**75+**
contacts with general practice	20.0*	19.1*	10.3*	8.9*	12.5*	16.3*	20.4*	25.4*
- face to face consultations	3.6*	3.4*	1.8	1.7	2.7*	3.2*	4.0*	3.5*
- home visits	1.8*	1.5*	0.8	0.1	0.4*	0.9*	1.5*	3.1*
prescriptions	19.5*	18.0*	5.1*	5.5*	8.5*	13.6*	19.7*	27.8*
referrals	0.50*	0.60*	0.27	0.26	0.52*	0.48*	0.60*	0.62*

* health care use of cancer patients is significantly higher compared to the health care use of the gender- and age-matched control group without cancer (figures of the control group not shown; p<0.01).

### Health care use and comorbid chronic conditions

The percentage of patients with at least one comorbid chronic condition was higher amongst the group of cancer patients compared to their matched controls (Table 3). This accounts for both men and women, but not for patients younger than 30 years of age.

**Table 3:** Percentage of cancer patients (n=8,703) and patients without cancer (n=8,672) with at least one comorbid chronic condition

	**cancer patients (%)**	**patients without cancer (%)**
- all	51.9*	44.5
- male	52.0*	43.9
- female	51.8*	44.9
Age group		
- ≤ 14 year	22.5	12.5
- 15–29 year	19.3	17.0
- 30–44 year	31.4*	24.2
- 45–59 year	42.1*	34.0
- 60–74 year	56.3*	50.2
- 75+ year	66.6*	58.1

* difference between cancer patients and patients without cancer: p<0.01.

Compared to the matched control group, GPs recorded heart disease, psychological, respiratory, skin and urogenital problems more often for cancer patients (Table 4). Endocrinological problems, amongst which diabetes, were the most frequent problems but they are not more frequently recorded in cancer patients than in the matched control patients.

**Table 4:** Percentage of cancer patients (n=8,703) and patients without cancer (n=8,672) with a comorbid chronic condition

	**cancer patients (%)**	**patients without cancer (%)**
Clusters of conditions:		
- sensory (including ear and eye)	1	1
- heart disease	19*	16
- musculoskeletal system	12	11
- neurological-movement	2	1
- neurological-pain	2	2
- psychological	10*	7
- respiratory	10*	8
- skin	8*	5
- endocrinological	21	19
- urogenital	6*	4

* difference between cancer patients and patients without cancer: p<0.01.

We split both groups (patients with and without cancer) in two according to the presence of comorbid chronic conditions (Table 5). Having cancer is related to significantly more health care use in general practice, irrespective of having a comorbid chronic condition. Patients with both cancer and a comorbid chronic condition have the highest use of care in general practice.

**Table 5:** Health care use of cancer patients and patients without cancer by the presence of a comorbid chronic condition (mean number of contacts, prescriptions and referrals per patient in a year)

	**No chronic condition**		**One or more chronic conditions**	
	**cancer patients (n=4,190)**	**patients without cancer (n=4,816)**	**cancer patients (n=4,513)**	**patients without cancer (n=3,856)**
contacts per year	14.4*	7.1	24.3*	18.0
- face to face consultations per year	2.6*	1.8	4.3*	4.0
- home visits per year	1.3*	0.2	2.0*	0.7
prescriptions per year	12.0*	5.6	24.9*	19.1
referrals per year	0.4*	0.2	0.7*	0.6

* difference between cancer patients and patients without cancer : p<0.01.

## Discussion

### Use of care in general practice

We found that during the follow-up phase cancer patients have double the use of health care services in general practice compared to controls. This included face to face contacts with GPs, home visits, prescribed medication and referrals to secondary specialist’s care. In the period after the end of initial treatment and before the start of the terminal phase, patients with cancer have more contacts with the general practice, amongst which more face to face consultations with the GP, more home visits, more prescriptions and they are more often referred to secondary care. They have more often one or more comorbid chronic conditions, amongst which heart, psychological, respiratory, skin and urogenital problems. Having a comorbid chronic condition further increases health care use in general practice.

In the literature, the few studies describing primary health care use of cancer patients report conflicting results [5-8,12,13]. One of the reasons is that health care systems and accessibility of secondary care differs between countries. Our study gives evidence that in the Dutch gatekeeper system, general practice has a substantial role in delivering care to cancer patients. This is in line with Roorda et al. [5] who reported more face to face contacts of breast cancer patients with GPs, more drug prescriptions and more referrals to secondary care during the year after the diagnosis compared to the year preceding the diagnosis.

### Comorbidity

Patients with both cancer and comorbid chronic conditions have the highest health care use in general practice. Comparable results were reported by Kurtz et al. [14] in the United States who found that use of primary care physicians' services depended substantially on comorbid conditions. Koroukian et al. [15] also reported that the complexity of health care demand increases when patients have cancer plus comorbid chronic conditions plus physical disability and/or geriatric syndromes. De Bock et al. [16] reported an increased prescription of psychotropic medication for female breast cancer patients on endocrine therapy. Anti-depressants were only prescribed during a short period, but anxiolytics, hypnotics and sedatives were prescribed for a much longer period. These data indicate increased psychological distress due to breast cancer diagnosis and/or treatment in women on endocrine therapy.

Several authors report that cancer survivors do not seem to receive the same quality of care for their comorbid conditions compared to the general population [12]. Examples are diabetes care [17], cardiopulmonary rehabilitation programs [18] or outpatient management of antithrombotic treatment [19,20]. Health care professionals seem to be more focused on cancer-related care compared to non-cancer related health care needs. Given the increase in the number of older patients who have more comorbid chronic conditions, this is a point of attention.

### Follow-up cancer care by GPs

We found that, in the Netherlands, health care use by cancer patients in general practice is quite substantial. It is expected that the role of GPs will increase further in the future. Cancer follow-up care is recognized internationally as a pressing health care issue. The number of patients is increasing, causing more strain on specialized in-hospital care. The benefit of follow-up in secondary care is questioned [21]. A more prominent role of GPs in follow-up care might come of help.

What do patients think of such a role for the GP? Patients often developed strong relationships with the specialist and staff members like nurses in the hospital during their treatment [22,23]. They hold the opinion that their GP is not sufficiently informed about their disease and treatment, and they did not want to bother their GPs with complaints [22]. However, Norwegian researchers found that both GPs and cancer patients valued the role of the GP in shared follow-up care as long as the specialist is easily accessible [24]. In several countries such initiatives arise, in which GPs join secondary care professionals in delivering follow-up care, so-called ‘shared care’. These initiatives can have different formats: from a scheduled consultation with the GP following the initial treatment [25] to a ‘shared care’ program in which specialists communicate extensively with GPs after discharge of the patient from the hospital and patients are stimulated to contact their GP in case of questions and problems [26]. To facilitate follow-up care for both GPs and patients a web-based survivor care plan might be used, including data on diagnosis, treatment and potential risks as well as recommendations for follow-up. In a recent study, such a web-based survivor care plan was positively evaluated by both patients and GPs [27].

Communication between specialist and GP is reported regularly as a major problem for the GP in delivering adequate care [28,29]. Standard information supplied by the specialist is often insufficient or too late. The coordination of patient care and communication between GPs and cancer specialists was found to improve when GPs are able to consult the electronic medical record of the specialist [30,31]. Furthermore, the development of guidelines, describing tasks and coordination in follow-up care for both specialists and GPs is of utmost importance [21].

### Limitations of the study

Because most patients are diagnosed with cancer in secondary care, the exact date of diagnosis is not always recorded appropriately in the EMR of the GP, neither is the date of the end (or start) of treatment. Therefore, we had to estimate the start of the follow-up phase: six months after the first recording of the diagnosis of cancer in the EMR. The date of the end of the observation period due to death or other reasons is registered accurately in the EMR, so we could exclude the terminal phase. The majority of cancer patients studied was still alive at the end of the study period. These facts combined, mean that the length of the follow-up phase studied differed for the cancer patients depending on time of inclusion, date of death or end of the study period.

## Conclusion

We found that cancer patients in the follow-up phase had higher health care utilisation in general practice compared to patients without cancer. It is expected that primary health care professionals will be involved even more in follow-up care for cancer patients in the future. When shifting the balance of follow-up care to general practice, attention is needed for the fact that many cancer patients have comorbid chronic conditions that need special attention. Consideration is then needed for coordination of tasks and communication between specialists and GPs.

## Competing interests

The authors declared that they have no competing interests.

## Author’s contribution

MvdA and FS designed the study and applied for funding. LJ contributed to the final design of the study, carried out the analyses and took the lead in writing the manuscript. FS and MvdA commented on draft versions of the manuscript. All authors read and approved the final version.

## Pre-publication history

The pre-publication history for this paper can be accessed here:

http://www.biomedcentral.com/1471-2296/13/60/prepub
